# Diarrhea, CD4 counts and enteric infections in a hospital – based cohort of HIV-infected patients around Varanasi, India

**DOI:** 10.1186/1471-2334-6-39

**Published:** 2006-03-01

**Authors:** Suresh VS Attili, AK Gulati, VP Singh, DV Varma, M Rai, Shyam Sundar

**Affiliations:** 1 Department of Medicine Institute of Medical Sciences, BHU, Varanasi-221005, India; 2 Microbiology Institute of Medical Sciences, BHU, Varanasi-221005, India; 3 Dermatology and STD clinic Institute of Medical Sciences, BHU, Varanasi-221005, India

## Abstract

**Background:**

As most of the studies in HIV patients with diarrhea were cross sectional, focusing on the etiological agents, we are reporting data on the rate of diarrhea, associations between diarrhea and CD4 counts and variation in frequency of identifying a pathogen with consistency of diarrhea and duration in a prospective hospital based study.

**Methods:**

Stool specimens were obtained between Jan 2001 and April 2003 from HIV infected adults with diarrhea presenting to Infectious Disease clinic, Banaras Hindu University, Varanasi. In all patients with diarrhea, specimens were examined by microscopy and cultures to identify pathogens.

**Results:**

During the study, 630 person years of observations with diarrhea were analyzed. 140 stool samples were collected representing 43% of episodes of reported diarrhea. Positivity of finding a pathogen from watery stools and formed stools were 40%&24% respectively (p < 0.01) probably due to associated inflammation is more in watery diarrhea. Patients having chronic diarrhea are 2.25 (95%CI 1.52–2.81) times at more risk of developing other opportunistic infections compared to those who don't have. However this is not true with the acute diarrhea where risk of harboring the opportunistic infections remain same.

**Conclusion:**

Diarrhea was most strongly associated with low CD4 counts. Over two-thirds of diarrheal episodes were undiagnosed, suggesting that unidentified agents or primary HIV enteropathy are important causes of diarrhea in this population. There is a strong negative association between duration of diarrhea and CD4 levels

## Background

HIV/AIDS is a major problem in India with more than 6 million cases by the end of 2005 [1]. HIV patients are prone to develop a panorama of diseases during their lifetime. Among them diarrhea is a significant cause of morbidity observed in majority of studies [2-5]. In fact it is the 2^nd ^leading cause of hospital visits in developing nations and makes its place in top 10 worldwide [1-11]. The information on the cause of diarrhea and the possibility of isolation of pathogens has largely come from various cross sectional studies [3-11]. Expectedly infectious etiologies lead the list in developing nations in contrast to non-infectious etiologies in developed nations. In most of these studies the emphasis was on chronic diarrhea, acute episodes and risk factors being largely unaddressed. As of now this data is available from two large prospective community based cohort studies that gave information on rates of diarrhea and isolation of enteric pathogens in developed nations [12,13]. These studies demonstrated a strong negative association between diarrhea and CD4 counts. Similarly they also found that majority of the samples didn't contain any pathogens, and wherever pathogens found, protozoan infections dominated over the bacterial causes. There are many reports regarding frequency of various pathogens causing diarrhea from different parts of India [3,4,14]. Some studies also demonstrated regional variability of pathogen [15], as well as changing trends of etiology in the same population (from infectious to non infectious) [16]. But reports regarding correlation of the diarrhea with CD4 levels, impact of CD4 levels on isolation of pathogen were not studied in India. The present study populations, North Indians form a distinct ethnic group owing to their typical dietary, living habits, low nutritional status, endemic E. histolytica infection and inherently low CD4 levels [2]. So we planned conduct a study to look for the rate of diarrhea, associations between diarrhea and CD4 counts and variation in frequency of identifying a pathogen with stool characters.

## Methods

All HIV infected patients attending the Infectious Disease (ID) clinic, Sir Sunderlal (SS) hospital were included in the study. SS hospital, affiliated to Institute of Medical Sciences, Banaras Hindu University is a tertiary care teaching hospital with catchments area of five states (UP, MP, Bihar, Jharkhand, and Chattisgarh) with 1000 beds. The annual attendance of new HIV cases is approximately 150. The study period was from Jan 2001 to April 2003. Stool specimens were obtained from HIV infected patients (aged from 12 years – 70 years) with diarrhea. An informed consent was taken from all the subjects of study as a routine workup (which includes consent to do HIV testing, CD4 estimation, and recording of the clinical details and for performing non invasive diagnostic tests.) which was already an existing practice in the ID clinic. A separate informed consent was taken from all the subjects of study prior to invasive procedures. The clinical diagnosis of AIDS was made by CDC criteria. In this population, HIV infection is transmitted mainly by heterosexual contact. The male female ratio was 3.7:1 with a median age of 34 years. The baseline characters of the patients were shown in the table 1 and the major symptoms of the patients were described in table 2.

**Table 1:** Baseline characters of the patients

Characters	Acute diarrhea (n = 102)	Chronic diarrhea (n = 111)	No diarrhea (n = 257)	P
Age	35.2± 12.6	38.9± 14.6	36.8+16.6	NS
Male female ratio	3.6:1	3.8:1	3.6:1	NS
Total duration of symptoms (in months)	11.6± 1.9	16.7± 2.9	10.8± 1.6	0.07
Mean time of AIDS diagnosis(in months)	4.57 ± 0.74	8.03 ± 0.513	2.74 ± 0.81	0.01
Mean CD4 levels	314.9	195.5	366.9	0.02
Patients on HAART	90 (88%)	96 (86%)	170 (66%)	0.07
Patients with AIDS (CDC definition)	100 (98%)	111 (100%)	207 (80%)	0.08
% with lower Socio economic status	68%	82%	79%	0.05

**Table 2:** Major symptoms in different CD4 groups

Symptom	CD4 <200 (n = 212)	CD4 200–500 (n = 117)	CD4 >500 (n = 46)
Weight loss	106	83	19
Fever	118	53	8
Diarrhea	74	57	8
Cough	48	47	12

### Definition of diarrhea

Symptoms reported by patients were used to classify diarrhea into acute and chronic episodes. Diarrhea was defined as the passage of two or more unformed stools in past 24 hr. An episode of diarrhea was classified as acute if it lasted for less than a month and provided the patient is diarrhea free in the preceding month. Episode was defined as chronic when diarrhea lasted more than a month, or was intermittent and recurrent over a period of at least two months with diarrheal symptoms for at least half this time. Sub-sequent diarrhea was classified as a new episode if there was a diarrhea-free interval of at least one month. If the duration of symptoms did not fit any of these definitions the case was excluded from the analysis.

### Sample collection

Stool samples were requested from patients who presented with diarrhea at the time of consultation as soon as possible. In cases wherever possible the patients were requested to collect the sample before starting any medication and bring it to our analysis. In patients, where samples were collected, 66% gave samples at the hospital. In the remaining patients, they were collected at home. Samples were collected in a wide mouth container at home and transported to us by the patients themselves. Due to poor access to transportation, in majority of the cases it took an average of 12 hours before the samples were brought to us. However we did not perform the routine bacterial cultures, owing to non feasibility (low education status, low motivation, economic and many other reasons) Information on the duration of symptoms of diarrhea was recorded in the case records maintained at ID clinic. In those cases where the information was incomplete the case were excluded from analysis.

### Microbiology

In all patients with chronic diarrhea, fresh fecal specimens from patients were examined directly. A small portion of the stool was emulsified in a drop of saline on a microscope slide and another portion in a drop of Lugol's iodine on another slide. These wet smears were examined under ×100, and ×400 magnifications for intestinal parasites. *Isospora belli oocyst *was easily identified as oval granular structures averaging 20–30 μ in length and10–20 μ in width. The presence of Isospora *& Cryptosporidium *oocysts was confirmed by examining the stool specimen by modified Ziel – Neelsen's stain. The smears (fixed in methanol for 1 minute) were flooded with 4% carbol fuchsin for 15 minutes without heating. Decolorizing was done by 1% acid alcohol for 5 minutes. Counter staining was done with 0.4%malachite green for 1 minute. Microscopic examination was carried out under low power, (×100), high power (×400) and oil immersion (×1000) lenses. Intense red, pink or faint pink, round or oval structures were especially searched for and brought to focus under higher magnification for confirmation and detailed study of their morphology. All specimens were subjected to stool cultures to identify bacterial causes.

### HIV serology

HIV – 1 status of the patients was confirmed by ELISA method using two different antigens [1].

### CD4/Viral load estimation

Immuno phenotype of lymphocytes was carried out by FACS count (Becton Dickinson, Singapore (BD). Viral load was not done due to economic constraints. CD4 counts were measured routinely in the first visit and during the follow-up visits. For this study, each time a participant provided a stool sample their most recent CD4 count, was used for the analysis. Stool samples from patients without CD4 information, were excluded from the analysis.

### Statistics

Analyses were performed using SPSS version 13.0 statistical software. The relationship between the CD4 count of the patient who provided the stool sample and duration of diarrheal symptoms were assessed using the t-test. A univariate analysis was performed to look at the association between organisms isolated and diarrhea. An adjusted analysis, using a logistic regression model, was then performed in to look at the association between organisms isolated and patients' CD4 counts (dichotomized in two groups: <200 and = 200 cells/μl).

## Results

**Figure 1:**
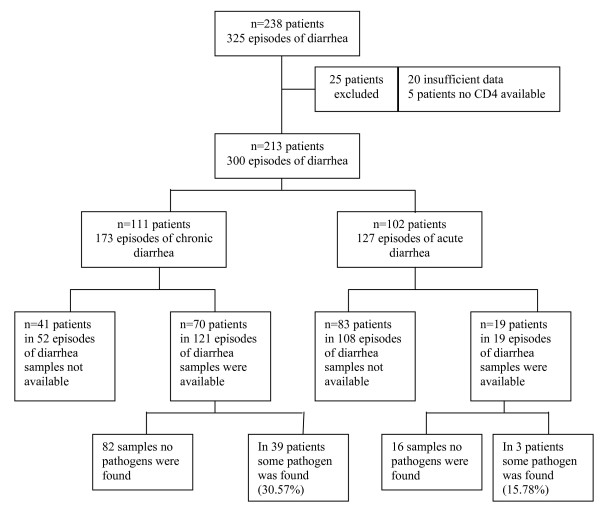


During this time 470 HIV-infected patients enrolled in the study, contributing 630 person years of observations (PYO). Clinical records were studied to determine the crude estimate of the rate of diarrhea. During this period, 238 patients made 415 clinic visits, with 325 distinct episodes of diarrhea, giving a crude rate of 849 diarrheal episodes (both acute and chronic) per 1000 PYO. During the study period 140 stool samples were collected, the breakdown of the diarrheal episodes the symptoms and reasons for exclusion are shown in figure 1.

The 140 samples from the 89 patients with diarrhea represented 43% of episodes of reported diarrhea. In the present study out of these140 samples, 100 samples were semi formed stools and 40 were watery. The positivity of finding a pathogen from watery stools and formed stools were 40% and 24 % respectively Those patients with diarrhea (acute or chronic) who gave stool samples were not different in terms of age, sex, or CD4 count to those others in the study who had diarrhea but from whom no stool sample was obtained (data not shown). The patients in whom pathogens were isolated were not at more risk of developing another opportunistic infection provided the nature of diarrhea (acute vs. chronic) remains same. The CD4 counts at enrollment were significantly lower for individuals who had chronic diarrhea, when compared to the participants who had an acute episode, data was shown in table 3 and 4.

**Table 3:** Relationship between cd4, symptoms and enteric pathogens.

**Causative Agent**	**No. of patients**	**CD4 Range**	**CD4 (Mean ± SEM)**	**% of stools containing organism**
E. Histolytica	24	28–186	112.35 ± 55.73	17.1%
Cryptosporidiosis	8	32–106	49.4 ± 22.6	5.71%
Isospora	4	65–70	67.5± 2.88	2.86%
Candida	4	80–85	82.5± 2.88	2.86%
Helminthes	2	155–125	140± 15	1.43%

**Table 4:** Unadjusted odds ratio, for the association between diarrhea, organisms and CD4 counts.

@	Unadjusted OR	95%CI		P**	Adjusted OR*(LR)	95%CI		P**
						
		MIN	MAX			MIN	MAX	
E. Histolytica	21.17	5.92	75.75	.000	46.91	8.69	'235.16	.000
Cryptosporidium	17.72	2.12	148	.001	29.94	2.67	336.04	.001
isospora	4.72	0.84	6.60	.075	15.11	2.22	102.62	.004
Candida	3.43	1.03	11.34	.050	4.74	0.91	24.56	.070
CD4 (<200/>200)	7.49	2.18	25.78	.000	1.94	.35	10.81	.434

@Percent isolates of above pathogens in healthy individuals were taken from the local reference values deducted in ***2000 ***in department of microbiology, I.M.S., BHU.

#CD4 levels of patients with and without identifiable pathogens in patients of diarrhea were compared

**P value of < 0.05 was taken as significant

Out of 140 stool samples collected in 300 episodes of diarrhea 42 protozoal pathogens were identified. When compared to the 8% incidence of the protozoal causes of chronic diarrhea in general population, it appears that the incidence of the protozoal etiology in HIV patients is more. In the remaining 98 episodes, 56 patients were on medications that were known to have diarrhea as one of the side effects (nelfinavir, amoxycillin, erythromycin etc...), however as the patients were taking these medications since long time further assumptions for its etiological role were not attempted.

### Cd4 counts and type of diarrhea

As at enrollment, CD4 counts among the study participants were significantly lower in those with diarrhea than in those without diarrhea. Patients with chronic diarrhea had lower CD counts than those with acute diarrhea. The data was shown in table 5.

**Table 5:** CD4 counts and type of diarrhea

**Type of diarrhea**	**No of Pt**	**Mean CD4**	**95% CI**		**P value**
				
			**min**	**max**	
Acute	111	314.9	259.7	370.1	<0.05
Chronic	102	195.5	170.1	220.9	<0.01
No diarrhea	162	366.9	305.1	428.7	

Patients having chronic diarrhea are 2.25 times at more risk of developing other opportunistic infections compared to those who don't have. However this is not true with the acute diarrhea where the risk of harboring the opportunistic infections remain the same.

### Durations of symptoms and CD 4 levels

**Figure 2:**
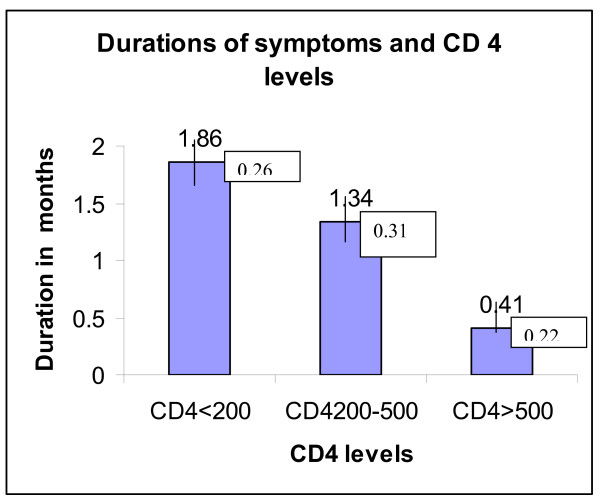


There was a strong negative association between the duration of diarrhea and CD4 levels (p < 0.05). The data was shown in Figure 2.

### Laboratory isolation of organisms

Pathogenic organisms were isolated from 42 stool samples (30%). Protozoa accounted for 85.7% (36/42) of all pathogens. E. histolytica was the most frequently encountered pathogen found in 24 stool isolates followed by opportunistic pathogens cryptosporidium spp. in 8 samples and Isospora in 4 samples. Candida was isolated in 4 diarrhea samples. All the patients, where Candida was isolated failed to respond to antibiotics but did show improvement with fluconazole. In two samples helminthes were isolated. Bacterial cause of diarrhea was not found in the present study. We did not study the viral cause of diarrhea. Ascaris, and hook worm were isolated in two patients with chronic diarrhea who also responded to albandazole therapy. Due to absence of facilities for diagnosis of microsporidiasis, which also respond to albandazole it was difficult make any comments on the causation of diarrhea by the helminthes.

## Discussion

Diarrhea is well recognized as an important component of HIV related morbidity. It has been included in the clinical case definition of AIDS by NACO & WHO [1,17]. In this hospital based study of HIV patients living in and round Varanasi, India, we tried to look for the etiology, risk factors, and rates of diarrhea. It has confirmed that diarrhea is common and there is a strong inverse association with CD4 counts particularly for chronic diarrhea. Similarly a few factors like CD4 levels, stool characters, and duration of diarrhea will affect the isolation rates of pathogens.

Majority of the study population were in advanced stage of HIV disease (AIDS), confirmed by clinical staging and CD4 level. In India the HIV prevalence in males is higher than females [1] as in the present study. Thus early HIV infection and females were under represented in the present study. Age, Sex, CD4 levels and clinical stage are similar in patients with diarrhea who did and did not give stool samples, suggesting no selection bias (therefore no disproportionate representation of particular group of individuals).

### Rate of diarrhea

Our rate of 849 diarrheal episodes per 1000 PYO is substantially greater than the 142 diarrheal episodes observed in the Swiss cohort, supporting the view that HIV-associated diarrheal disease occurs more commonly in India [12]. Our rate represents a minimum burden of diarrhea, as episodes were only counted if a patient came to the clinic. Diarrhea which was self-limiting or which responded to over-the-counter medication would not have come to our attention.

### Isolation rates

Rates of identification of organism from stool sample were less compared to other studies [2-5,8-24],. The pathogens were more frequently associated with chronic rather than acute diarrhea. Despite using the best possible diagnostic techniques, the isolation rates did not exceed 34–73% in chronic diarrheas and the results were much depressing in acute diarrhea where the isolation were 14–51% [3-5]. Our results are towards the lower ranges reported in the literature.

### Effect of CD4

It was observed that the CD4 cell count influenced the cause of diarrhea as well as the diagnostic yield [25]. The diagnostic yield of stool analysis is low in patients with higher CD4 cell counts. The probable reasons as felt by the authors was

1. Effective HAART helps eradicating opportunistic protozoal infection, and associated with the influx of CD4 positive cells into the lamina propria.

2. As the opportunistic infections causing diarrhea in AIDS become less common, other gastrointestinal diseases, which are common in young age group, like inflammatory bowel disease and coeliac disease, irritable bowel syndrome and idiopathic steatorrhoea are presently leading the list of etiological agents.

3. Variety of unknown/unidentified infections or HAART-related toxicities.

### Local factors

It is observed in the present study that 28.9% of chronic and 15.7% of acute diarrheal episodes were experienced by patients with CD4 counts of = 200 cells/μl, indicating that it is a frequent problem even in less advanced stages of HIV. One of the reasons could be regional immunosuppression as suggested by Schneider et al [26]. They found, loss of CD4 cells in intestinal mucosa of the patients with diarrhea, which were more pronounced than peripheral CD4 levels and their relation is quite variable. The mucosal immunity, an important factor to prevent diarrhea is therefore variable even in patients with good immunity (i.e. peripheral CD4>200). We did not perform the mucosal CD4 levels thus any comments on the mucosal immune status based on blood CD4 levels would be in appropriate. But we can presume that probably the low mucosal immunity could be a cause of diarrhea in patients with high CD4 levels.

### Stool characters

There was considerable variation in the isolation of pathogens from formed and watery diarrhea [3].The probability of finding a pathogen from watery and formed stools were 42% and 16% respectively in present study not very different from the literature (40&24%). As most of the studies did not take stool characteristics into consideration, it is difficult to compare the present study with other studies. However there is little doubt that nature of stool specimen examined bears a good correlation (p < 0.01) in identifying a pathogen, watery diarrhea giving a better yield of organism than a semi formed stool. The greater positivity could be due to greater shedding, more inflammatory response and greater virulence of the pathogens causing watery diarrhea.

### Comparison with other studies

As compared to the literature, the results of the present study had substantially lower positivity rates and low rates of infections. One of the major reasons is that the present one is prospective nature and hospital based study compared to others which were cross sectional data and laboratory based. The comparison was represented in table 6. The low positivity in the present study could be because of many other reasons as well.

**Table 6:** Results of the present study compared to other studies

**Organism**	**Langewer et al^4 ^(1996) (n = 77)**	**Joshi et al^3 ^(2002) (n = 110)**	**Mohandas et al^14 ^(2002) (n = 36)**	**Present study (2003) (n = 140)**
E.histolytica	-	14.9%	1.7%	17.1%
Cryptospora	22.8%	8.5%	10.8%	5.71%
Isospora	4.8%	17%	2.5%	2.86%
Candidacies	-	-	-	2.86%
Other	-	4.3%	22.8%	1.43%
Strongyloides stercoralis	1.2%	5.3%	-	-

1. Widespread community use of drugs, (co-trimoxazole, metronidazole etc..), for the treatment of diarrhea prior to hospital visit.

2. Low education status leading to non production of sample till patient becomes symptom free.

3. More percent of formed stools in the present study

4. Unavailability of diagnostic facilities for microsporidiasis and viral causes.

As none of our patients had fever, neutrophils in the stool sample or other features of acute inflammation, probability of a bacterial cause is less likely, though in presence of the immunosuppression these features could often be absent. However we don't deny improper sample collection and transportation for the same. In 65 of 82 samples of patients with chronic diarrhea, and in 3/16 patients with acute diarrhea the patients were on co- trimoxazole for P. carnii prophylaxis which might be responsible for at least some cases of pathogen negative diarrhea. A well designed study, with proper sample collection and transportation, might enlighten us in this aspect.

### Pathogen negative diarrhea

Pathogen negative diarrhea is likely to include cases caused by organisms not identified in this study, by the HIV virus itself and perhaps to a lesser extent by malignancies.

### Common antibiotic prescription

The role of antibiotics in the management of diarrhea is currently unclear, particularly in those individual whose diarrhea is chronic and not self-limiting. Current recommendations of giving co-trimoxazole in chronic diarrhea based on the frequency of Isospora are probably inappropriate in this population. However in view of the frequent E histolytica infections, which still are sensitive to the co-trimoxazole it can still, be continued. But in the areas were resistance to co-trimoxazole was documented, metronidazole will be a better alternative.

## Conclusion

1. North Indian population had higher diarrheal episodes(849PYO)

2. Protozoal etiologies dominate over others in this population

3. Watery stools had a better diagnostic yield

4. Strong inverse relation was observed between acute diarrhea, short duration of symptoms and pathogen negative diarrhea with CD4 levels.

## Pre-publication history

The pre-publication history for this paper can be accessed here:


